# Supplementary material for: Associations between youth’s daily social media use and well-being are mediated by upward comparisons
Source: Commun Psychol. 2023 Aug 22;1:12. doi: 10.1038/s44271-023-00013-0 (PMC11332017; doi:10.1038/s44271-023-00013-0)
Supplement: Supplementary file 2 — Supplemental Information [file 44271_2023_13_MOESM2_ESM.pdf]

Supplementary Information

for

Associations Between Youth's Daily Social Media Use and Well-Being are Mediated by Upward Comparisons

by

Andrea Irmer[1] (née Schmidt) & Florian Schmiedek[1,2]

published in

Communications Psychology

[1] DIPF | Leibniz Institute for Research and Information in Education, and Center for Research on Individual Development and Adaptive Education of Children at Risk (IDeA), Frankfurt am Main, Germany

[2] Goethe-University, Frankfurt am Main, Germany

Supplementary Table 1

Model Fit of the Measurement Models of Interest

Model	χ^2	Scaling factor	*df*	RMSEA	CFI	$SRMR_{within}$	$SRMR_{between}$	AIC	BIC
				Positive and Negative Self-Worth					
1-factor	1374.76	1.99	40	.12	.57	.14	.11	40,217.68	40,448.66
2-factor	209.46	1.82	38	.04	.95	.03	.03	37,868.56	38,111.08
				Positive and Negative Affect					
1-factor	856.21	1.17	28	.11	.64	.08	.08	36,898.95	37,101.08
2-factor	76.36	1.39	26	.03	.98	.03	.03	36,004.03	36,217.71
				Upward Social Comparisons					
1-factor	108.72	3.21	18	.05	.93	.04	.04	27,225.80	27,399.06

Note. *df* = degrees of freedom; RMSEA = Root Mean Square Error of Approximation; CFI = Comparative Fit Index; SRMR = Standardized Root Mean Square Residual; AIC = Akaike Information Criterion; BIC = Bayesian Information Criterion

Supplementary Table 2

Multilevel Structural Equation Models: Examining Potential Cross-Level Interaction Effects of Person-Level Variables

Effect	Outcome				
	Upward Comparisons	Positive Self-Worth	Negative Self-Worth	Positive Affect	Negative Affect
Within-Person Effects					
Upward Comparisons		**-0.26 [-0.31, -0.20]**	**0.27 [0.22, 0.33]**	**-0.25 [-0.30, -0.19]**	**0.29 [0.23, 0.34]**
Between-Person Effects					
Upward Comparisons		**-0.61 [-0.70, -0.50]**	**0.69 [0.59, 0.77]**	**-0.57 [-0.67, -0.45]**	**0.67 [0.56, 0.76]**
Sex		0.03 [-0.12, 0.18]	-0.07 [-0.21, 0.08]	0.08 [-0.08, 0.24]	-0.06 [-0.22, 0.10]
Cross-Level Interaction Effect					
Upward Comparisons x Sex		-0.03 [-0.33, 0.26]	-0.06 [-0.35, 0.25]	0.16 [-0.13, 0.42]	-0.27 [-0.52, 0.03]
Within-Person Effects					
Social Media Use	**0.09 [0.04, 0.13]**	**-0.08 [-0.13, -0.04]**	**0.06 [0.02, 0.11]**	-0.03 [-0.08, 0.02]	0.04 [-0.00, 0.09]
Between-Person Effects					
Social Media Use	**0.38 [0.25, 0.50]**	**-0.27 [-0.40, -0.13]**	**0.21 [0.07, 0.34]**	**-0.31 [-0.44, -0.17]**	**0.26 [0.11, 0.39]**
Sex	**0.21 [0.04, 0.38]**	-0.10 [-0.27, 0.08]	0.08 [-0.09, 0.26]	-0.04 [-0.21, 0.14]	0.10 [-0.09, 0.27]
Cross-Level Interaction Effect					
Social Media Use x Sex	0.23 [-0.07, 0.51]	-0.13 [-0.44, 0.18]	0.08 [-0.22, 0.35]	-0.17 [-0.44, 0.10]	0.05 [-0.23, 0.34]
Within-Person Effects					
Upward Comparisons		**-0.26 [-0.31, -0.20]**	**0.27 [0.21, 0.33]**	**-0.24 [-0.29, -0.18]**	**0.26 [0.21, 0.32]**
Between-Person Effects					
Upward Comparisons		**-0.59 [-0.69, -0.48]**	**0.69 [0.59, 0.77]**	**-0.54 [-0.65, -0.41]**	**0.68 [0.57, 0.77]**
Self-Control Failure		-0.04 [-0.16, 0.08]	-0.03 [-0.15, 0.09]	-0.06 [-0.19, 0.07]	-0.05 [-0.18, 0.07]
Cross-Level Interaction Effect					
Upward Comparisons x Self-Control Failure		-0.02 [-0.26, 0.21]	0.05 [-0.19, 0.30]	-0.00 [-0.22, 0.22]	0.05 [-0.18, 0.28]

Within-Person Effects					
Social Media Use	**0.09 [0.04, 0.14]**	**-0.08 [-0.13, -0.03]**	**0.07 [0.02, 0.12]**	-0.03 [-0.08, 0.01]	0.04 [-0.01, 0.09]
Between-Person Effects					
Social Media Use	**0.32 [0.18, 0.45]**	**-0.23 [-0.37, -0.08]**	**0.17 [0.01, 0.31]**	**-0.26 [-0.41, -0.12]**	**0.24 [0.08, 0.39]**
Self-Control Failure	**0.20 [0.07, 0.33]**	**-0.14 [-0.29, -0.001]**	0.12 [-0.03, 0.27]	-0.14 [-0.28, 0.01]	0.08 [-0.07, 0.22]
Cross-Level Interaction Effect					
Social Media Use x Self-Control Failure	0.05 [-0.20, 0.29]	-0.09 [-0.33, 0.17]	-0.12 [-0.36, 0.13]	-0.03 [-0.24, 0.20]	-0.02 [-0.26, 0.23]
Within-Person Effects					
Upward Comparisons		**-0.26 [-0.32, -0.21]**	**0.27 [0.21, 0.33]**	**-0.25 [-0.30, -0.20]**	**0.27 [0.21, 0.33]**
Between-Person Effects					
Upward Comparisons		**-0.58 [-0.68, -0.47]**	**0.67 [0.57, 0.75]**	**-0.54 [-0.65, -0.41]**	**0.64 [0.53, 0.73]**
Social Comparison Orientation: Opinion		-0.10 [-0.22, 0.02]	0.04 [-0.08, 0.15]	-0.08 [-0.12, 0.05]	0.07 [-0.60, 0.19]
Cross-Level Interaction Effect					
Upward Comparisons x Social Comparison Orientation: Opinion		0.12 [-0.13, 0.34]	-0.01 [-0.25, 0.23]	**0.23 [0.002, 0.43]**	-0.02 [-0.25, 0.23]
Within-Person Effects					
Social Media Use	**0.09 [0.05, 0.14]**	**-0.08 [-0.13, -0.04]**	**0.07 [0.02, 0.11]**	-0.03 [-0.08, 0.02]	0.04 [-0.01, 0.09]
Between-Person Effects					
Social Media Use	**0.35 [0.23, 0.47]**	**-0.24 [-0.37, -0.10]**	**0.18 [0.03, 0.31]**	**-0.28 [-0.41, -0.14]**	**0.23 [0.08, 0.36]**
Social Comparison Orientation: Opinion	**0.26 [0.13, 0.38]**	**-0.25 [-0.38, -0.11]**	**0.22 [0.08, 0.35]**	**-0.21 [-0.34, -0.07]**	**0.24 [0.10, 0.37]**
Cross-Level Interaction Effect					
Social Media Use x Social Comparison Orientation: Opinion	0.06 [-0.18, 0.28]	-0.01 [-0.24, 0.24]	-0.12 [-0.35, 0.12]	-0.15 [-0.35, 0.07]	-0.05 [-0.28, 0.18]
Within-Person Effects					
Upward Comparisons		**-0.26 [-0.32, -0.21]**	**0.27 [0.21, 0.32]**	**-0.25 [-0.31, -0.19]**	**0.27 [0.21, 0.33]**

Between-Person Effects					
Upward Comparisons		**-0.56 [-0.66, -0.44]**	**0.64 [0.53, 0.73]**	**-0.52 [-0.64, -0.39]**	**0.64 [0.52, 0.73]**
Social Comparison Orientation: Ability		-0.12 [-0.25, 0.00]	0.10 [-0.03, 0.21]	-0.09 [-0.23, 0.04]	0.06 [-0.07, 0.20]
Cross-Level Interaction Effect					
Upward Comparisons x Social Comparison Orientation: Ability		0.09 [-0.14, 0.31]	0.03 [-0.20, 0.27]	0.09 [-0.13, 0.30]	-0.00 [-0.22, 0.23]
Within-Person Effects					
Social Media Use	**0.09 [0.05, 0.14]**	**-0.09 [-0.13, -0.04]**	**0.07 [0.02, 0.11]**	-0.04 [-0.09, 0.01]	0.04 [-0.01, 0.09]
Between-Person Effects					
Social Media Use	**0.31 [0.18, 0.43]**	**-0.20 [-0.34, -0.06]**	0.13 [-0.02, 0.27]	**-0.25 [-0.39, -0.11]**	**0.19 [0.05, 0.33]**
Social Comparison Orientation: Ability	**0.31 [0.18, 0.43]**	**-0.28 [-0.42, -0.15]**	**0.31 [0.17, 0.44]**	**-0.23 [-0.36, -0.08]**	**0.27 [0.12, 0.40]**
Cross-Level Interaction Effect					
Social Media Use x Social Comparison Orientation: Ability	0.05 [-0.19, 0.27]	0.12 [-0.12, 0.35]	-0.10 [-0.34, 0.14]	0.09 [-0.12, 0.30]	-0.00 [-0.23, 0.23]

Note. Table displays standardized parameter estimates and 95% credible interval in parentheses. Parameters whose credible interval does not include zero are highlighted in bold. For each moderator, three models were calculated. In the first model, social media use predicted each of the four subjective well-being indicators, in the second model, social media use predicted upward social comparisons, and in the third model, upward social comparisons predicted each of the four subjective well-being indicators. Sex was entered as a dichotomous categorical variables with 0 = males and 1 = females.

Supplementary Discussion

Newly Developed Measures

Within the scope of the present study, we developed scales assessing daily upward social comparisons, daily negative self-worth, and daily subjective social media use in children and young adolescents. Within- and between-person reliabilities of upward social comparisons and negative self-worth were acceptable (within: .83, .82; between: .96, .96). For the social media use scale, we adopted a formative measurement model assuming the manifest indicators to cause the latent construct (instead of a reflective measurement model assuming the latent construct to cause the manifest indicators). Therefore, it was not sensible to determine the reliability of the scale in terms of internal consistency. However, different work[1] shows that the scale showed good convergence with an objective measure of social media use.

We assessed self-worth as a two-dimensional construct with a positive and a negative part. The inclusion of a negative part besides the positive part was motivated by previous work[2], suggesting that positive self-worth represents only one side of possible attitudes towards the self and that low agreement with positive statements (i.e., low satisfaction with oneself or low feeling of pride) is not equivalent to negative attitudes (i.e., being disappointed by oneself or wishing to be different). However, the dimensionality of self-worth (or self-esteem) has been a topic of debate in the existing literature[3,4]. For instance, in a meta-analysis, Huang and Dong[3] found a two-factor structure of self-esteem (positively vs. negatively worded items) to be supported in all studies except for one. Nonetheless, as the discriminant validity of the two factors was low, the authors concluded that "viewing these two factors as entirely different constructs may be unnecessary unless they have different correlates"[3 (p.136)]. The present work contributes to this debate, as we also found a two-dimensional model to represent the data better than a one-dimensional model. Examining the associations between social media use and self-worth as well as the associations between upward social

comparisons and self-worth, we did not find differences in the significance of effects on positive vs. negative self-worth. Hence, in this matter, we did not identify different correlates as suggested by Huang and Dong[3]. Yet, in the mediation model, only the direct within-person effect of social media use on negative self-worth became significant, while the direct within-person effect of social media use on positive self-worth still was significant. Furthermore, only the total effect of social media use on negative self-worth was significant, while the total effect of social media use on negative self-worth was not. Altogether, however, there were no major differences in the effects on positive vs. negative self-worth.

Supplementary References

1. Irmer A and Schmiedek F. How accurately do children indicate their smartphone social media use? A comparison of subjective and objective reports in children's everyday lives. *Z Psychol* (2023, in press).

2. Schmidt A, Dirk J, Neubauer AB, and Schmiedek F. Evaluating sociometer theory in children's everyday lives: Inclusion, but not exclusion by peers at school is related to within-day change in self-esteem. *Eur J Pers* **35**, 736–753. DOI: 10.1177/0890207020962328, (2021).

3. Huang C and Dong N. Factor structures of the Rosenberg Self-Esteem Scale. *Eur J Psychol Assess*, **28**, 132–138. DOI: 10.1027/1015-5759/a000101, (2012).

4. Marsh HW, Scalas LF, and Nagengast B. Longitudinal tests of competing factor structures for the Rosenberg Self-Esteem Scale: Traits, ephemeral artifacts, and stable response styles. *Psychol Assess*, **22**, 366–381. DOI: 10.1037/a0019225, (2010)
