# Supplementary material for: Associations between youth’s daily social media use and well-being are mediated by upward comparisons
Source: Commun Psychol. 2023 Aug 22;1:12. doi: 10.1038/s44271-023-00013-0 (PMC11332017; doi:10.1038/s44271-023-00013-0)
Supplement: Supplementary file 3 — Reporting Summary [file 44271_2023_13_MOESM3_ESM.pdf]

nature portfolio

Corresponding author(s): Andrea Irmer & Florian Schmiedek

Last updated by author(s): Jul 13, 2023

Reporting Summary

Nature Portfolio wishes to improve the reproducibility of the work that we publish. This form provides structure for consistency and transparency in reporting. For further information on Nature Portfolio policies, see our Editorial Policies and the Editorial Policy Checklist.

Statistics

For all statistical analyses, confirm that the following items are present in the figure legend, table legend, main text, or Methods section.

n/a	Confirmed	
☐	☒	The exact sample size (*n*) for each experimental group/condition, given as a discrete number and unit of measurement
☐	☒	A statement on whether measurements were taken from distinct samples or whether the same sample was measured repeatedly
☐	☒	The statistical test(s) used AND whether they are one- or two-sided<br>*Only common tests should be described solely by name; describe more complex techniques in the Methods section.*
☒	☐	A description of all covariates tested
☒	☐	A description of any assumptions or corrections, such as tests of normality and adjustment for multiple comparisons
☐	☒	A full description of the statistical parameters including central tendency (e.g. means) or other basic estimates (e.g. regression coefficient) AND variation (e.g. standard deviation) or associated estimates of uncertainty (e.g. confidence intervals)
☐	☒	For null hypothesis testing, the test statistic (e.g. *F*, *t*, *r*) with confidence intervals, effect sizes, degrees of freedom and *P* value noted<br>*Give P values as exact values whenever suitable.*
☐	☒	For Bayesian analysis, information on the choice of priors and Markov chain Monte Carlo settings
☐	☒	For hierarchical and complex designs, identification of the appropriate level for tests and full reporting of outcomes
☐	☒	Estimates of effect sizes (e.g. Cohen's *d*, Pearson's *r*), indicating how they were calculated

Our web collection on statistics for biologists contains articles on many of the points above.

Software and code

Policy information about availability of computer code

Data collection	Data were collected via self-report questionnaires implemented on soscisurvey.de
Data analysis	We used Mplus Version 8.8 (Muthén & Muthén, 1998-2017) to analyze our data. Data and analysis code necessary to reproduce the results reported are available in the Open Science Framework at https://osf.io/cs9um/.

For manuscripts utilizing custom algorithms or software that are central to the research but not yet described in published literature, software must be made available to editors and reviewers. We strongly encourage code deposition in a community repository (e.g. GitHub). See the Nature Portfolio guidelines for submitting code & software for further information.

Data

Policy information about availability of data

All manuscripts must include a data availability statement. This statement should provide the following information, where applicable:

- Accession codes, unique identifiers, or web links for publicly available datasets
- A description of any restrictions on data availability
- For clinical datasets or third party data, please ensure that the statement adheres to our policy

A detailed study protocol, a codebook including the original German items with English translations, and data and analysis code necessary to reproduce the results reported here are available in the Open Science Framework at https://osf.io/cs9um/.

Research involving human participants, their data, or biological material

Policy information about studies with human participants or human data. See also policy information about sex, gender (identity/presentation), and sexual orientation and race, ethnicity and racism.

Reporting on sex and gender	We reported on sex, which was reported by parents of participating children. There participated 200 children in our study, parents were asked to indicate their sex (0 = male, 1 = female, 2 = non-binary). The German language does not make the same distinction between sex and gender as English, which is why it is more likely that parents reported on their child's biological sex as compared to their gender. Therefore, we used the word "sex" throughout our mansucript. No parent reported that their child was non-binary, which is why the sex variable was dichotomous in the present study. We collected informed consent from all participants and parents. We reported sex-based moderation analyses in the manuscript and supplement and discussed on the findings in the manuscript.
Reporting on race, ethnicity, or other socially relevant groupings	Parents of participating children reported what type of school their child attended (i.e., primary school, the academic tier of secondary school, ...) as well as their current employment status. This information is provided in the manuscript. Further information can be found in the study protocol that is available in the Open Science Framework https://osf.io/cs9um/.
Population characteristics	See above
Recruitment	The present data were collected in Germany between April 6, 2021 and June 4, 2021. Information on study details were disseminated via social media platforms (e.g., the Instagram accounr of the authors' institution), e-mails to schools and the federal parents' council, sport and music clubs, and word-of-mouth marketing. We provide this information in the manuscript. We also discuss on possible selection biases in the Discussion. Although invitation letters were sent out to all types of schools, primarily principals of the academic tier of secondary school forwarded our emails to the families of the target classes.
Ethics oversight	The study was approved by the Ethics committee of the DIPF | Leibniz Institute for Research and Information in Education (DIPF_EK_2021_11).

Note that full information on the approval of the study protocol must also be provided in the manuscript.

Field-specific reporting

Please select the one below that is the best fit for your research. If you are not sure, read the appropriate sections before making your selection.

☐ Life sciences ☒ Behavioural & social sciences ☐ Ecological, evolutionary & environmental sciences

For a reference copy of the document with all sections, see nature.com/documents/nr-reporting-summary-flat.pdf

Behavioural & social sciences study design

All studies must disclose on these points even when the disclosure is negative.

Study description	The study was a 14-day diary study embedded within a pre and post assessment. Surveys were implemented on soscisurvey.de as online questionnaires. Data were quantitative.
Research sample	Two-hundred children and young adolescents (103 girls) between the ages of ten and 14 years (Mage = 11.71, SDage = 1.02) and one of their parents (163 mothers) participated in this study. Inclusion criteria for study participation were that children owned a smartphone with Internet access and were able to understand the German language. Most of the participating youths (n = 151, 75.5%) attended the academic tier of secondary school (Gymnasium) and had German as their native language (n = 160, 80.0%). At the time of the assessment, 84.5% (32.5%) of fathers (mothers) were employed full-time, 10.0% (56.0%) were employed part-time, and 4% (10.0%) were unemployed. We collected a convenience sample of children and young adolescents in Germany. Although invitation letters were sent out to all types of schools, primarily principals of the academic tier of secondary school forwarded our emails to the families of the target classes. Consequently, the sample was positively selected (i.e., high education and high income), limiting the generalizability of findings. We discuss on this in the manuscript.
Sampling strategy	The present data were collected in Germany within the zEbra study between April 6, 2021, and June 4, 2021. Information on study details were disseminated via social media platforms (e.g., the Instagram account of the authors' institution), e-mails to schools and the federal parents' council, sport and music clubs, and word-of-mouth marketing. Participation was voluntary. We did not perform a power analysis; however, the overall compliance rate was good (85%) and slightly higher than in comparable studies (Heron et al., 2017), yielding 2382 available data points out of a maximum of 2800 data points.
Data collection	Information on study details were disseminated via social media platforms (e.g., the Instagram account of the authors' institution), e-mails to schools and the federal parents' council, sport and music clubs, and word-of-mouth marketing. comprised four parts: a parental questionnaire, a baseline questionnaire, a 14-day diary period, and a post questionnaire. All assessments were implemented as online questionnaires on soscisurvey.de. In the first part, parents were asked to complete a background questionnaire (approximately 10 minutes) assessing their child's native language, number of siblings, and their child's personality, for instance. Then, children filled in the baseline questionnaire that started with a video in which we explained the study procedure and instructed participants in how to respond to the items. After having watched the video, children were asked to respond to items on their typical social media use, personality, and well-being, for instance. Completing this part of the study took about 30 minutes. The following

	day, the 14-day diary period began. During this time, children received a daily e-mail at 7pm with a link to an online questionnaire. They could access the questionnaire daily from 7pm to 10pm and were instructed to complete it (which took about ten minutes) just before bedtime. The day after the diary part of the study, children received a link to a post questionnaire containing similar items as the baseline questionnaire, additional measures such as pathological social media use or a questionnaire on emotion regulation problems, as well as feedback on study participation. This final questionnaire took approximately ten minutes to complete. More detailed information can be found in the study protocol available in the Open Science Framework at https://osf.io/cs9um/.
Timing	Data were collected between April 6, 2021 and June 4, 2021.
Data exclusions	No data were excluded.
Non-participation	No participant dropped out during the study.
Randomization	No experimental design, no randomization to groups.

Reporting for specific materials, systems and methods

We require information from authors about some types of materials, experimental systems and methods used in many studies. Here, indicate whether each material, system or method listed is relevant to your study. If you are not sure if a list item applies to your research, read the appropriate section before selecting a response.

Materials & experimental systems

n/a	Involved in the study
☒	☐ Antibodies
☒	☐ Eukaryotic cell lines
☒	☐ Palaeontology and archaeology
☒	☐ Animals and other organisms
☒	☐ Clinical data
☒	☐ Dual use research of concern
☒	☐ Plants

Methods

n/a	Involved in the study
☒	☐ ChIP-seq
☒	☐ Flow cytometry
☒	☐ MRI-based neuroimaging
